# Targeting Human α-Lactalbumin Gene Insertion into the Goat β-Lactoglobulin Locus by TALEN-Mediated Homologous Recombination

**DOI:** 10.1371/journal.pone.0156636

**Published:** 2016-06-03

**Authors:** Hongmei Zhu, Jun Liu, Chenchen Cui, Yujie Song, Hengtao Ge, Linyong Hu, Qian Li, Yaping Jin, Yong Zhang

**Affiliations:** 1 College of Veterinary Medicine, Northwest A&F University, Yangling, 712100, Shaanxi, China; 2 Key Laboratory of Animal Biotechnology, Ministry of Agriculture, Northwest A&F University, Yangling, 712100, Shaanxi, China; Imperial College London, UNITED KINGDOM

## Abstract

Special value of goat milk in human nutrition and well being is associated with medical problems of food allergies which are caused by milk proteins such as β-lactoglobulin (BLG). Here, we employed transcription activator-like effector nuclease (TALEN)-assisted homologous recombination in goat fibroblasts to introduce human α-lactalbumin (hLA) genes into goat BLG locus. TALEN-mediated targeting enabled isolation of colonies with mono- and bi-allelic transgene integration in up to 10.1% and 1.1%, respectively, after selection. Specifically, BLG mRNA levels were gradually decreasing in both mo- and bi-allelic goat mammary epithelial cells (GMECs) while hLA demonstrated expression in GMECs in vitro. Gene-targeted fibroblast cells were efficiently used in somatic cell nuclear transfer, resulting in production of hLA knock-in goats directing down-regulated BLG expression and abundant hLA secretion in animal milk. Our findings provide valuable background for animal milk optimization and expedited development for agriculture and biomedicine.

## Introduction

Human α-lactalbumin (hLA), the main whey protein in human milk, accounting for 41% of the whey and 28% of the total protein, has a primary function in regulating the synthesis of lactose which plays an important role in milk production[1, 2]. hLA contains a high proportion of the essential amino acids of tryptophan and cysteine[3, 4]. Due to its balanced nutrient, α-lactalbumin has been added as a new bioactive ingredient in infant formula[5]. Since it can indirectly activate the galactose to the receptor sugar glucose with high specificity, hLA is thus considered to be a valuable constituent of diets for patients whose protein intake must be restricted. In addition to the function of increasing iron absorption and exerting bactericidal activity in the neonatal digestive track[6, 7], the complex of hLA with oleic acid can also induce tumour cell apoptosis and be confirmed effective for prevention and treatment of colon cancer [8–11].

β-Lactoglobulin (BLG), which is scarce in human breast milk, accounts for 53.7% of the total whey protein in goat milk and is a primary milk allergen that causes infant hypersusceptibility[12, 13]. The presence of 2 disulfide bonds is suspected to be responsible for BLG allergic effects[14]. At present, goat milk and its by-products of yoghurt, cheese and powder have great significance in human nutrition and are widely appreciated around the world[15, 16]. Several approaches such as heat treatment, enzymatic hydrolysis, fermentation and glycation have been applied to reduce the allergenic potential of BLG protein. However, these methods are generally cost and may affect other valuable nutrients. Thus, to extend the use of goat milk as a nutrient for human beings or to “humanize” goat milk, we applied TALENs to knock out BLG gene and simultaneously increase hLA component to improve the nutritional quality of goat milk, which is unlike the prime pharmaceutical interest to generate animal bioreactors in livestock[17, 18].

Homology recombination (HR) which faithfully restores the original sequence by copying it from the sister chromatid for repairing double-strand breaks (DSBs) is low in its frequency for precise genomic modification. Engineered endonucleases including zinc-finger nucleases (ZFNs), transcription activator-like effector nucleases (TALENs) and RNA-guided DNA endonucleases (RGEN) are programmable genome editing tools that can generate DSBs at preferred genomic regions and improve HR efficiency by several orders of magnitude[19]. ZFNs have been applied in cattle production to disrupt the BLG gene[20]. TALENs mediated disruption of BLG and insertion of human lactoferrin in BLG locus have also been successfully produced in our previous study[21]. Though TALENs have been widely used in gene-targeting research in cows, pigs[22], and human somatic and pluripotent stem cells[23], the production of genetically modified goat with hLA inserted in BLG locus using TALENs has not been reported.

Since a TALEN pair corresponding the exon1 of BLG locus had been efficiently applied in our previous report, our objective here is to utilize this gene-targeting system in goat fibroblasts by inserting the exogenous hLA gene into the BLG locus and subsequently using the targeted cell clones as donors for somatic cell nuclear transfer (SCNT). Considering that production of transgenic animals requires vast numbers of surrogates, immense labour and substantial funding, the precise identification of targeted clones, evaluation of tissue-specific expression of the transgene in goat mammary epithelial cells (GMECs) and assessment of the reconstructed embryos in vitro were carried out in present research. Milk assays of transgenic founder were performed to verify the feasibility for our procedure. Furthermore, to ensure that the genetic modification can be transmitted by germline, the gene targeting analysis for F_1_ offspring was carried out in current study.

## Materials and Methods

### Ethics statement

All experiments were approved by the Care and Use of Animals Centre, Northwest A&F University. This study was performed in strict accordance with the Guidelines for the Care and Use of Animals of Northwest A&F University. Goat ovaries were collected from the Tumen abattoir, a local slaughterhouse in Xi’An, P.R. China. Recipient goats were obtained from Yangling Keyuan Cloning Co., Ltd. Every effort was made to reduce the number of animals used, and all surgeries were performed under anaesthesia via the intravenous injection of Sumianxing, a compound anaesthetic containing dimethylaniline thiazole, dihydroetorphine hydrochloride, ethylenediaminetetraacetic acid, and haloperidol (0.01 mL/kg; Veterinary Research Institute, Jilin, China).

### Generation of the targeting vector pLoxpII-hLA-neo and a TALEN pair

**Fig 1:**
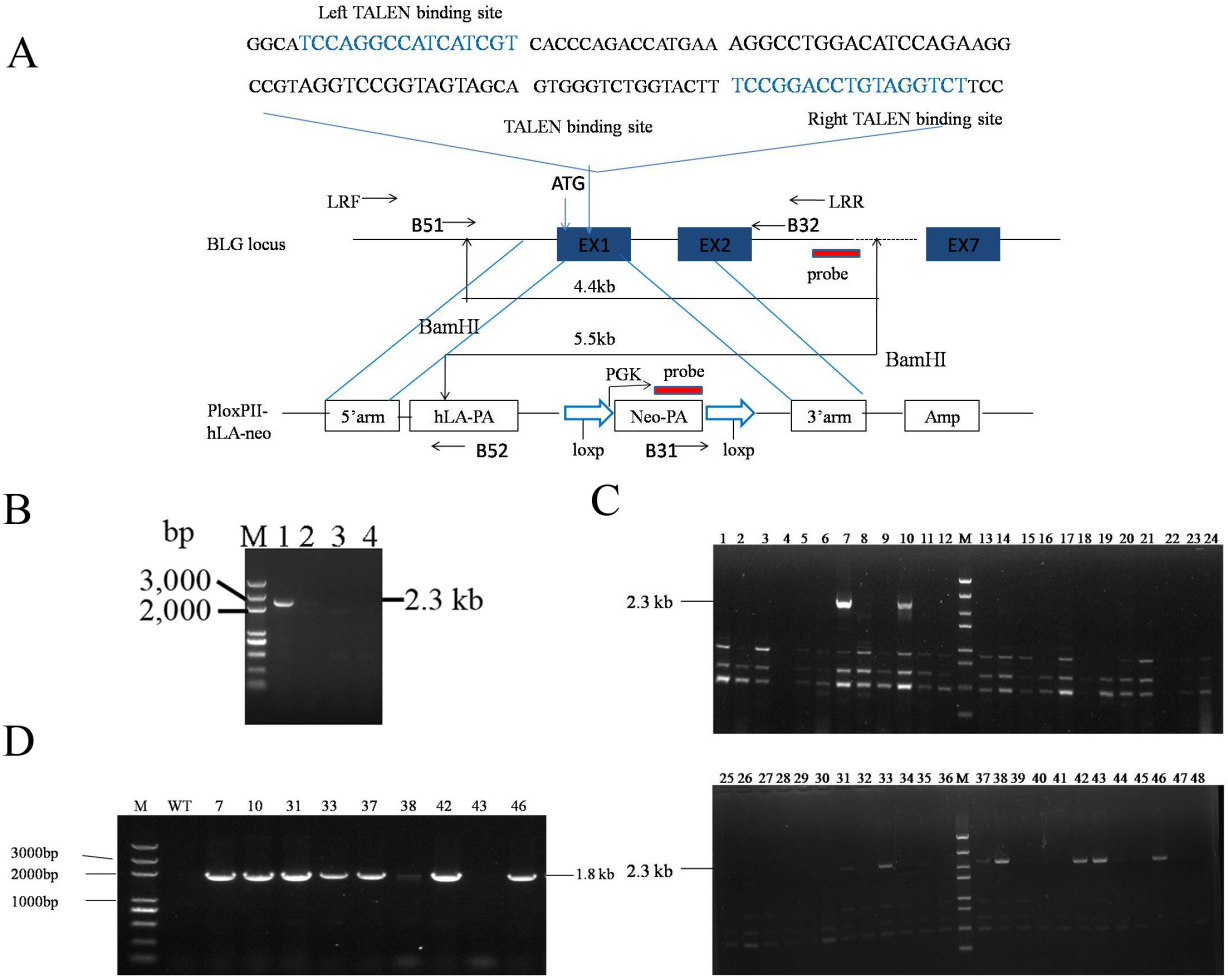
Targeting BLG in goat fibroblasts. (A) Schematic overview depicting the targeting strategy for the BLG locus. Blue boxes, exons of BLG; Diagonal lines above, TALEN1/2 pair binding sites; Level arrow lines, primers used for PCR; The predicted size of southern hybridization bands with BamHI digestion, for both the endogenous BLG locus and the BLG targeted locus, is indicated. (B) PCR-based measurements of TALEN-driven exogenous gene integration into the BLG locus in goat fibroblasts. Cells were right untransfected (lane 4, for negative control) or were transfected with an expression cassette for TALENs that induce a DSB at exon1 of BLG locus (lane 3), and donor plasmids carrying a foreign gene flanked by two homology arms, in the absence (lane 2) and presence (lane 1) of the TALEN. (C) 3’ Junction PCR results from cell lysis templates. TALEN-mediated transgene insertion yielded 2,300-bp PCR products using primers B31 and B32, which were specific to the neo gene and the BLG locus, respectively. (D) 5’ Junction PCR analysis performed on genomic DNA of 3’ junction PCR-positive colonies using primers B51 and B52 to amplify the 1,800-bp left-hand junction between the endogenous BLG locus and the exogenous hLA.

The two homologous arms 5’ (738-bp) and 3’ (714-bp) were produced by PCR amplification from the goat genome. The 2,100-bp hLA DNA sequences (from signal peptide to stop codon) were acquired from the human blood genome. The neomycin resistance gene (neo), driven by a phosphoglycerol kinase (PGK) promoter, was designed for positive clone selection and was flanked by two loxP sites which would resulted in the removal of neo sequence by cre-mediated recombination. The hLA-neo cassette was flanked by homologous arms in the PloxpII-hLA-neo vector (Fig 1A). A TALEN1/2 pair targeting exon1 of the BLG gene (GenBank: Z33881) was designed as our previous study[21]. Briefly, the binding sites were selected by the “TAL Effector-Nucleotide Targeter”[24] and assembled by the “unit assembly” method[25]. The assembled TALEN vectors were linearized by NotI to be used as templates for the in vitro TALEN mRNA transcription with the AmpliCap^™^ SP6 High Yield Message Maker Kit (Epicentre).

### Transfection and selection of the primary cells

The goat fetal fibroblasts (GFFs) and goat ear fibroblasts (GEFs) were established from 40-day-old foetuses and the ear skin of approximately two-month-old kids, respectively. The early-passage (P1-P3) primary fibroblast cell lines were used for transfection with the BTX Electro cell manipulator ECM2001 (BTX Technologies). The plasmid pLoxPII-hLA-neo (12 μg) and the two TALEN plasmids or mRNA (8 μg) were co-electroporated into cell suspensions under the condition of 510 V, 2 ms, and 1 pulse. Transfected cells were then seeded into 90-mm plates with DMEM/F12 amended with 15% FBS under 37°C in a 5% CO_2_ environment. 48 h later, the cells were screened with 700 mg/ml G418 (Sigma). G418-resistant cell clones were picked into a 48- or 24-well cell culture plate according to clone size and cell concentration.

### Detection of gene insertion by PCR analysis

A small part cells of each clone were resuspended in 15–20 μL cell lysis buffer (40 mM Tris-HCL, pH 8.9; 0.9% Triton X-100, 0.9% Nonidet P-40, 0.4 mg/ml proteinase K[26]). The lysates were then incubated at 65°C for 15 min and heated to 95°C for 10 min. 2–4 μL DNA lysates were used as template in junction PCR reaction with primers B31/B32 (3’ end, 2,300-bp product) or with primers B51/B52 (5’ end, 1800-bp product). The genomic DNA of each cell colony was extracted using the TIANamp genomic DNA kit (Tiangen Biotech) for long-range PCR with LA Taq polymerase (TaKaRa); The purified PCR fragments above were cloned into the pMD19-T vector (TaKaRa) and sent for sequencing (GenScript Co., Ltd). Primers used for targeting analysis are presented in S1 Table.

### Expression analysis on the mRNA and protein levels in vitro

The GMECs were screened with 500 mg/ml G418 (Sigma). A TranScript First-Strand cDNA Synthesis SuperMix Kit (Transgen) was used for reverse-transcription PCR analysis on total RNA samples. The primers BcF/BcR and hcF/hcR which were specific for BLG and hLA partial cDNAs were shown in S1 Table. Supernatants from the induced mammary epithelial cells were collected every 12 hours. The recovered supernatants were then vacuum freeze-dried and subjected to western blot analysis. The primary rabbit anti hLA antibody (1:1,000) used to detect hLA was from Santa Cruz Biotechnology.

### Nuclear transfer

The SCNT procedure was performed according to a previously described report[27]. Briefly, the Cumulus–oocyte complexes (COCs) were cultured to maturation for 22–24 hours. The first polar bodies and chromosomes of the oocytes were suctioned out and the targeted donor cells were injected into the perivitelline space of the enucleated oocytes. Karyoplast-cytoplast couplets were fused by electrofusion. The couplets were incubated for 2–3 h in TCM-199. Fused embryos were activated in 5 mM ionomycin for 4 min and then cultured in 2 mM 6-dimethylaminopyridine in mSOF medium for 4 h. The embryos were then washed extensively and transferred into 200 μL mSOF supplemented with 10% FBS while covered with mineral oil at 38.5°C, 5% CO_2_.

### Southern blot analysis

Southern blot analysis was performed according to standard procedures with the DIG High Prime DNA Labelling and Detection Starter Kit II (Roche). A 3’ external hybridization probe (800-bp) and a neo probe (500-bp) were labelled for gene insertion (Fig 1A). The diagnostic fragments should were a 5.5-kb BamHI fragment extending across the 3’ arm of the targeted allele and a 4.4-kb BamHI fragment of endogenous DNA extending across the 3’ and 5’ arms at the site of the wild-type allele.

### Isolation of whey protein in goat milk

Milk samples were centrifuged at 10,000 g for 15 min to remove the fat fractions and diluted with an equal amount of PBS. The primary fat-free supernatant liquids were then adjusted to pH 3.8–4.6 with 1 M HCl and centrifuged at 10,000 g for 15 min to eliminate the casein fraction. The secondary casein-free supernatant liquids were then adjusted to pH 6.8 with 1 M NaOH for SDS-PAGE detection and western blot analysis.

### Statistical analysis

Data presented are derived from at least three independent experiments. Statistical significances were analyzed using the One-Way ANOVA. A value of P<0.05 was considered significant.

## Results

### Generation of targeting vector PloxpII-hLA-neo

Based on the target site of TALENs (Fig 1A), we generated a targeting vector PloxpII-hLA-neo to introduce hLA-neo cassette to exon 1 of the goat BLG locus. Two homology arms flanking hLA-neo cassette were designed for introducing HR which can repair the DSBs induced by TALEN pair. Upon the successful integration, the 90 bp fragment containing the ATG start codon to partial exon1 sequence of the BLG gene would be deleted and replaced with the hLA-neo cassette, which would result in BLG protein loss and hLA expression driven by endogenous BLG promoter.

### Preparation of gene-targeting clones with TALENs

To investigate the efficient TALEN-induced exogenous gene integration into BLG locus, the targeting vector PloxpII-hLA-Neo (S1 Fig), along with TALEN plasmids were engineered to introduce a DSB in exon1 of BLG gene in goat fibroblasts (Fig 1A). After 48 h of culturing the cells in normal medium without any drug selection, we harvested genomic DNA and measured the integration of the exogenous gene by 3’ junction PCR. As expected, no measurable exogenous gene integration into the chromosome was observed in the absence of TALENs. By contrast, the junction region between endogenous and exogenous DNA was amplified by primers B31 and B32 in cells that were exposed both to the donor plasmid and TALENs (Fig 1B; Lane 1).

Approximately 4×10^6^ cells containing GFFs and GEFs were transfected with targeting plasmids and the TALEN pair (Table 1). A total 741 colonies were obtained from four transfections with 10 days of G418 selection (700 mg/ml). To exclude the random DNA integration, the DNA lysates from each clone were used for the initial 3’ junction PCR (Fig 1C). Products with a size consistent with this process were readily detected in 13.5% (n = 741) of the PCR samples. Subsequent 5’ junction PCR was performed to amplify the left-hand junction between the endogenous BLG gene and hLA DNA (Fig 1D). As evidence of this fact, approximately 88% of the 3’ junction PCR-positive clones were confirmed correct during this procedure (Table 1).

**Table 1:** Efficiency of gene targeting in goat somatic cells using TALENs.

Cell lines (sex)	No.of G418^R^ colonies	No.of 3’Junction PCR^+^ colonies (%)	No.of 5’Junction PCR^+^ colonies (%)	No.of LR-PCR^+^colonies (%)	No.of bi-allelic targeted colonies (%)	Senesced^a^	Targeted coloniessuitable for NT
GEF1(F)	194	25(12.9)	21(10.8)	19(9.79)	2(1.03)	3	16
GEF9(F)	200	23(11.5)	21(10.5)	21(10.5)	2(1.0)	5	16
GFF3(F)	158	29(18.4)	27(17.1)	26(16.5)	3(1.89)	8	18
GFF8(F)	189	23(12.2)	19(10.05)	17(9.0)	1(0.53)	5	12
Total	741	100(13.5)	88(11.9)	83(11.2)	8(1.1)	21	62

^a^Colonies were scored as senesced when cell numbers were not observed to increase seven days after seeding.

### Screening of BLG bi-allelic targeted clones from that of mono-allelic clones

**Fig 2:**
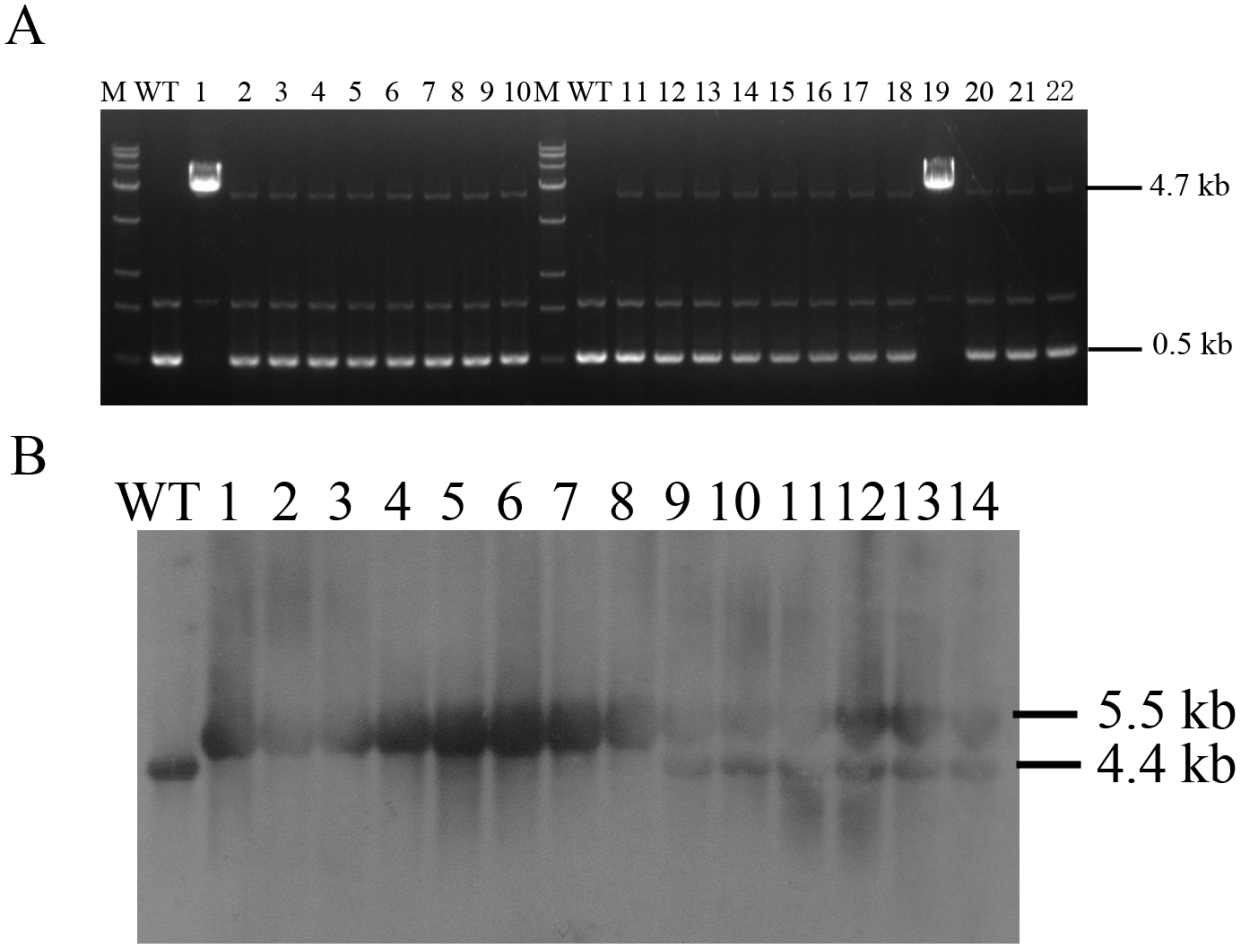
Identification for BLG bi-allelic modification clones. (A) Long-range PCR analysis performed on genomic DNA by primers LRF and LRR. Lane WT, non-transgenic cells; Lanes 1–22, gene-targeted clones. The wild-type allele PCR product was 0.5 kb and the targeted allele PCR product was 4.7 kb. (B) Southern blot analysis for gene targeted clones. Lane WT, non-transgenic cells; Lanes 1–8, BLG bi-allelic modification clones identified by LR-PCR; Lanes 9–14, BLG mono-allelic modification clones identified by LR-PCR.

The long-range (LR) PCR primers LRF and LRR, located at the recombination site, were used to confirm correct targeting and to exclude false positive clones. We can see that in some clones there were only 4.7-kb targeted bands existing without endogenous 0.5-kb bands (Fig 2A; Lanes 1 and 19), this may illustrated that these clones are targeted at two alleles. Southern blot analysis demonstrated that these clones were targeted at two alleles of BLG gene (Fig 2B; Lanes 1–8). Totally, we found mono- and bi-allelic frequency of 10.1% and 1.1%, respectively, in all G418-resistant colonies through LR-PCR analysis (Table 1).

### Expression of the hLA in goat mammary epithelial cells in vitro

**Fig 3:**
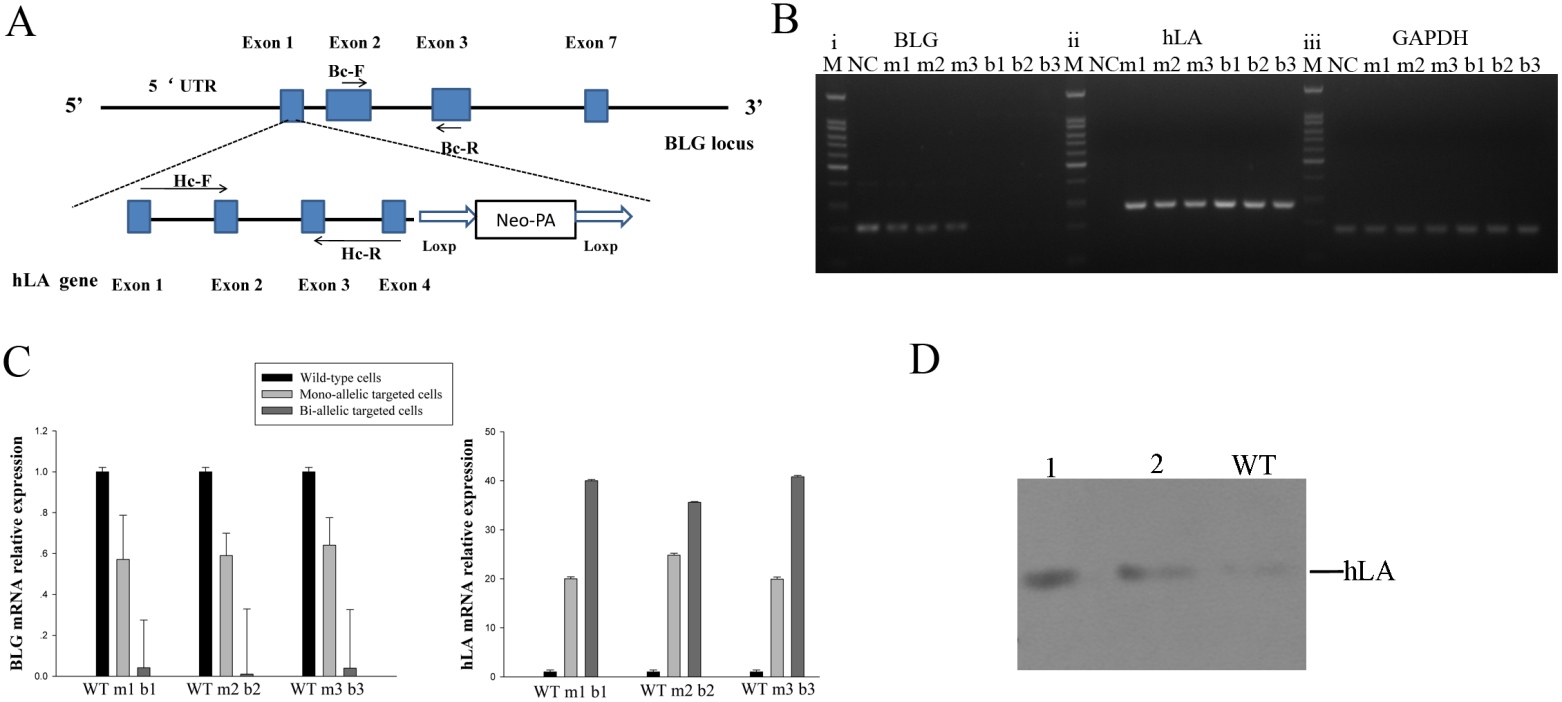
Human α-lactalbumin gene expression in GMECs in vitro. (A) Shematic overview dipicting position of primers Bc-F/Bc-R and Hc-F/Hc-R on BLG and hLA exons respectively. Blue boxes, exons of BLG or hLA; Level arrow lines, primers used for RT-PCR. (B) Reverse-transcription PCR analysis of partial BLG (i), hLA (ii) and GAPDH (iii) mRNAs in mono- and bi-allelic BLG-targeted cell clones. NC: non-transfected cells; m1, m2, m3: mono-allelic BLG-targeted clones; b1, b2, b3: bi-allelic BLG-targeted cell clones. (C) qPCR analysis of BLG and hLA expression. WT: non-transfected cells; m1, m2, m3: mono-allelic BLG-targeted clones; b1, b2, b3: bi-allelic BLG-targeted cell clones. The results were derived from at least three different experiments, P<0.05. (D) Western blot analysis for hLA in the supernatants of transfected GMECs. Lane WT, supernatant of non-transfected cells; Lanes 1–2, supernatant of bi-allelic and mono-allelic BLG-targeted cell clones.

To verify that BLG expression levels was reduced by hLA insertion and the endogenous BLG promoter was driving expression of hLA, the targeting construct and the TALEN pair were co-transfected into GMECs by FugenHD. Screening GMEC clones with 500 mg/ml G418 and then performed PCR analysis for gene insertion. PCR-positive GMECs were treated with inductive medium (DMEM/F12 amended with 10 μg ml^-1^ EGF, 1% ITS, 5 μg ml^-1^ prolactin, and 1 g ml^-1^ hydrocortisone). Simultaneously, non-transfected cells were cultured in the same condition as controls. After 48–72 h of induction, total RNA was extracted from the experimental cells; hLA and BLG partial cDNAs were successfully generated from RT-PCR on the total RNAs (Fig 3A and 3B). Quantitative real-time PCR (qPCR) was performed to detect BLG and hLA gene expression levels in single clone at the transcriptional level. BLG mRNA level was down-regulated by 36.0%-43.0% and 99.6–99.9%, respectively, in BLG mono-allelic and bi-allelic knock-in clones. Moreover, hLA exhibited high mRNA expression levels both mo-allelic and bi-allelic modification clones (Fig 3C). To further confirm mammary-specific expression of the hLA transgene at the protein level, the supernatants of transfected GMECs treated with the inductive medium were collected and processed via western blot analysis (Fig 3D).

### Production of gene-targeted goats by SCNT

**Fig 4:**
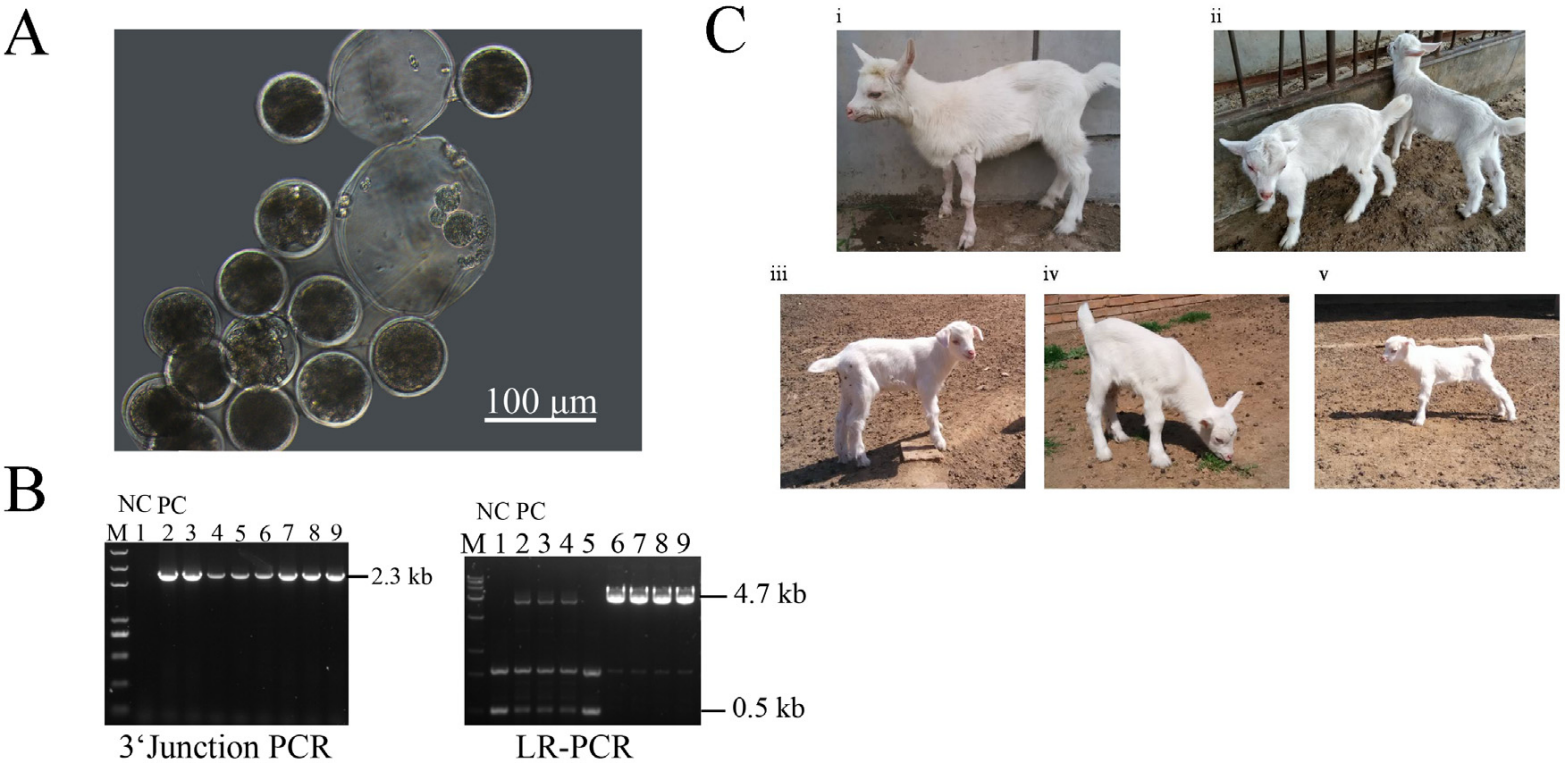
Goat gene-targeted cloned embryos developed in vitro. (A) The goat reconstructed embryos cultured in vitro. (B) Seven nuclear cloned embryos deriving from different clones were used for individual-embryo PCR assays to rule out mixed colonies that containing non-targeted cells. Lane 1, non-transgene cloning embryo used for negative control; Lane 2, gene-targeted cell colony used for positive control. Lanes 3–9, reconstructed embros. (C) The gene-targeted goats postpartum (born may 2014).

To eliminate the potential integration of TALEN–DNA constructs on genome, we applied TALEN-encoding mRNAs to generate targeted cells to be used as donors for SCNT. In total, PCR analysis showed the targeting efficiency of 8.35% (n = 1032) by TALEN-mRNA mediated foreign gene integration (S2 Table). Subsequently, We have carefully examined several most potential off-target sites of this TALEN pair in the gene-edited fibroblasts via sequencing before SCNT, and no detectable off-target mutations were found in the genome of the clones (S3 Table). A total of 4 mono-allelic hLA knock-in colonies (GEF21, GEF94, GEF97, GFF86) and 1 bi-allelic hLA knock-in clone (GFF31) (Table 2) with normal chromosome numbers, compact spindle-like cell morphology, and rapid growth were considered suitable for SCNT. The in vitro developmental experiment was carried out to evaluate blastocyst formation after SCNT (Fig 4A). In order to rule out mixed colonies (colonies contained non-targeted cells), 7 cloned embryos were used for 5’ junction PCR and long-range PCR analysis. Fig 4B showed that there were heterozygous or homozygous embryos for the hLA gene knock-in at the BLG locus, and thus we would have one or two targeted copies of the BLG gene. Ultimately, 1112 embryos were reconstructed, and 717 embryos developing to 2–4 cells were surgically transferred to 50 recipients (Table 2). Seventeen pregnancies were detected by ultrasonography at day 60, and five were carried to term, resulting in six live births (Fig 4C). The birth weight for six cloned goats is shown in S4 Table, the one bi-allelic targeted goat deriving from GFFs died soon after one month due to pneumonia and anemia, other five goats deriving from GEFs have grown to adulthood. The other pregnancies were lost for spontaneous abortion or unknown reason.

**Table 2:** Nuclear transfer of gene-targeted goat somatic cell lines.

Nuclear donor cell line	No.of embryoscultured	No.of embryosTransferred (%)^a^	No.of recipients	No.of pregnants (%)^b^	No.of kids born(%)^c^
GEF-21	224	138(69.3)	10	5(50.0)	2(1.45)
GEF-94	200	130(65.0)	9	3(33.3)	1(0.77)
GEF-97	217	135(62.2)	10	3(33.3)	2(0.92)
GFF-31	220	147(67.0)	10	2(20.0)	1(0.68)
GFF-86	251	167(66.9)	11	4(36.4)	0

^a^Developmental rate = number of embryos transferred / number of embryos cultured.

^b^Pregnancy rate = number of pregnant recipients / number of recipients used.

^c^Cloning efficiency = number of kids born / number of embryos transferred.

**Fig 5:**
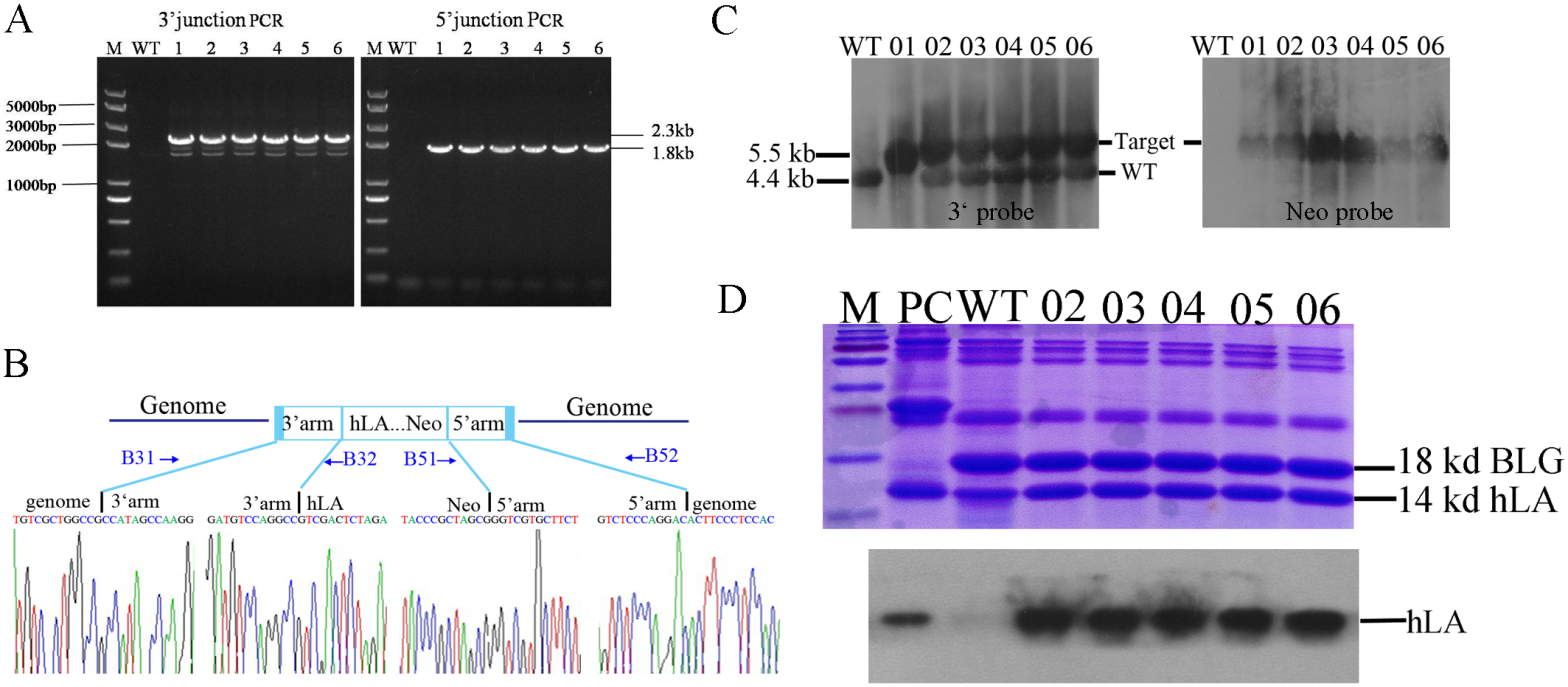
Analysis of knock-in goats. (A) Junction PCR analysis. Lane WT, wild-type goat; Lanes 1–6, transgenic goats. (B) Nucleotide sequence analysis of the junctions between the endogenous and exogenous DNAs corresponding to HR events. The 1,800-bp and 2,300-bp PCR products specific for the left- and right- hand junctions are indicated by the peak figure below respectively. (C) Southern blot analysis. Lane WT, wild-type goat; Lane 01, bi-allelic targeted goat; Lanes 02–06, mono-allelic targeted goats. (D) Analysis of milk from mono-allelic BLG-targeted goats. (top) Analysis of whey proteins by coomassie blue staining after separation by 15% SDS/PAGE. Equal amounts of milk samples were loaded onto each lane of the gel; (bottom) Western blot analysis of hLA expression. PC, human milk as positive control; WT, wild-type goat; Lanes 02–06, mono-allelic targeted goats.

Finally, junction PCR screening for each locus revealed integration of the transgene in the cloned goats (Fig 5A). DNA sequencing indicated that the 90-bp BLG fragment had been replaced with the exogenous 4.2-kb fragment of hLA-neo cassete. There were no point mutations or micro-rearrangements, such as base substitutions or insertions/deletions, on either side of the two homology arms either (Fig 5B). Southern blot analysis of the genomic DNA from these cloned goats suggested that five goats harboured one targeted allele and one harboured a two-allele insertion (Fig 5C).

### Analysis of the milk from animals carrying the hLA allele

To investigate whether the recombinant hLA can secrete in goat milk, five pubertal BLG^hLA/+^ goats were crossed with wild-type goats for natural lactation. After parturition, milk whey proteins from BLG^hLA/+^ goats were analyzed by 15% SDS gel electrophoresis and Coomassie blue staining. As shown in Fig 5D in the upper panel, the 18-KD BLG bands from targeted goat milk were weaker than that of wild-type goat, this illustrate that BLG reduced in transgenic goat’s milk. As shown by western blot analysis in the lower panel, hLA was highly expressed in BLG^+/hLA^ goat milk compared with that of normal goat. The results indicate that the insertion of hLA in BLG locus is disrupting BLG expression and endogenous BLG promoter is driving the hLA expression in transgenic goat milk. As measured with an enzyme-linked immunosorbent assay (ELISA), the hLA concentration for BLG^hLA/+^ goat milk was 1.2 mg/ml. A milk composition analysis of fat, protein, lactose and milk solids revealed insignificant differences between transgenic and non-transgenic goats (Table 3).

**Table 3:** Comparison of the components of milk from transgenic versus non-transgenic animals. No significant differences were detected between the two groups with respect to every component (p>0.05).

Animas	Fat(%)	Protein(%)	Lactose(%)	Solid(%)
Transgenic(n = 3)	3.74±0.24	3.92±0.23	4.99±0.19	12.33±0.48
Non-transgenic(n = 3)	3.90±0.37	3.34±0.15	4.50±0.22	12.30±0.35

The BLG^+/hLA^ heterozygous goats were normal in appearance and behaviour. Seven F_1_ offspring were monitored via PCR and southern blot analysis (S2 Fig). As expected, two progenies were inherited one mutant allele from their mothers (S2 Fig and S5 Table). These data indicated that the site-specific insertion for exogenous hLA in BLG locus is heritable.

## Discussion

In the current study, we demonstrate the feasibility of a TALEN-stimulated gene addition at the endogenous BLG loci and the successful production of cloned goats by SCNT. Our usage of TALENs, LR-PCR for bi-allelic targeting identification and in vitro hLA expression evaluation resulted in an effective procedure for preparing transgenic donor cells for SCNT. Milk production of transgenic goats will enable the direct evaluation of the allergenicity of BLG protein as well as the full investigation of hLA biological function in animal or human experiments.

Novel analogous technologies such as ZFNs, TALENs and RGEN system are programmable, site-specific engineering technologies enabling precise manipulation in natural chromosomal contexts. A previous study on the mouse Rosa26 gene demonstrated that specific TALENs exhibit superior targeting efficiency to that of ZFNs specific for the same targeting sequence[28]. Furthermore, ZFNs suffering from cytotoxicity and limited target sites[29, 30] have restricted their applications. For RGEN technique, a rising number of researches have revealed high potential off-target cleavage due to three or fewer mismatches in their guide RNAs and the difficulty of controlling the concentrations of Cas9:guide RNA complexes [31, 32]. These data document the superiority of TALENs to other two gene editing tools in gene-insertion applications at present.

TALEN-mediated isolation of colonies with mono- and bi-allelic modification by non-homologous end-joining (NHEJ) around cleavage sites or by HR in livestock has been reported [22, 33]. Nevertheless, few studies report bi-allelic LR-PCR analysis in targeted clones selection [26, 34, 35]. In general, time-consuming backcrossing of founder goat or sequential gene targeting with another targeting construct into the second allele is performed. Undoubtedly, LR-PCR analysis for the efficient detection of double-allelic clones based on exogenous integration between two arms and DNA cleavage site in our study reduced the time and labour costs. Furthermore, distance between the homology arms and the DNA cleavage site can affect TALEN-induced HR efficiency. In this report, the efficacy of HR using homologous arms contiguous to the DSB site (~64 bp for 5’arm and ~10 bp for 3’arm) was comparative to that in previous study when the homology was ~17 bp or ~80 bp from the DSB site in vitro[21]. These results further suggested that the precise TALEN-mediated deletion or insertion with DNA templates may have more extensive application when refer to DSB sites.

Though foetal fibroblasts are ideal donors for cloning livestock [26, 35, 36], the weakness for this selection is the inability to confirm the presence of heritable diseases in the foetus. As shown in Table 2, our method revealed a superior cloning efficiency of GEFs (0.92%-1.45%) than GFFs (0–0.68%). In present study, we found that the GEF cells have a more typical fibroblast morphology and a smaller cell individuals than GFF cells before or after G418 selection (S3 Fig). This may result in higher cloning efficiency of GEFs and demonstrate that GEFs are excellent substitute for GFFs for cloning goats.

To avoid the occurrence of silencing or aberrant expression of transgene[37, 38], the inductive in vitro expression of exogenous transgene in GMECs was performed. BLG expression reduction by almost 36–43% and 99% in mono- and bi-allelic targeted GMECs, rather than the expected 50% and 100%, in vitro, was consistent with our previous speculation that the expression of the intact BLG allele was up-regulated to compensate for the missing allele in mono-allelic targeted goats[21]. Our observation of high hLA expression in BLG^+/hLA^ goat milk indicates that all elements necessary for high α-lactalbumin expression are contained within the endogenous BLG fragment. High hLA expression may also balance milk protein synthesis. A water increase caused by lactose may be responsible for the insignificant change on lactose content between transgenic goats and non-transgenic goats. This would in return indicate that the volume of transgenic goats has risen in transgenic goats. Our data in Table 3 revealed that hLA insertion have not influence the mammary system and milk secretion mechanism balance itself well in transgenic goats.

Compared to the hLA random integration in goat genome and being driven expression specifically in goat mammary gland by synthetic BLG promoter[18], our study introduced the hLA gene into specific BLG locus and exploited the endogenous BLG regulatory fragment to direct abundant hLA secretion in goat milk as well as disrupt BLG expression. The current procedure is superior in avoiding position effect, important unknown endogenous gene silencing and the transgenic animal safety issue which may be caused by random integration [39, 40]. Furthermore, rather than simply adding hLA protein without reducing other components in goat’s milk which would affect the balance of milk composition[41], the BLG protein loss in present research can be complemented by hLA secretion. Further experiments on milk safety and functional analysis have to be carried out in future. Importantly, the germline transmission of targeting mutations offers us a opportunity for mass production of targeting animals.

In conclusion, we have demonstrated that TALEN-mediated HR can be used to create both mono- and bi-allelic insertion in fibroblasts, leading to the production of BLG mono- and bi-allelic targeted goats directing reduced BLG content and abundant hLA secretion in goat milk. The production of this goat strain is great progress for people who are allergic to goat or cow milk. We also expect that hLA presence in transgenic goat’s milk could exhibit its function of increasing milk production and protecting the body from bacterial invasion as well as tumorigenesis in future study. We anticipate that our success on germline-transmittable production of hLA knock-in goats through TALEN-mediated genome engineering would expand the field of goat milk application in health care and therapeutic supplies.

## Supporting Information

S1 Fig Plasmid map of targeting construct PloxpII-hLA-neo. A donor plasmid was created corresponding to the cleavage location of the TALEN pairs, and each side carried approximately 730-bp regions of homology to the BLG sequence astride the cleavage site.
(DOC)

S2 Fig Analysis of F1 offspring. (A) 5’Junction PCR analysis on seven F1 offspring. WT, wild-type goat; Lanes 1–7, seven F1 offspring. (B) 3’Junction PCR on two offspring identified targeted from 5’junction PCR. WT, wild-type goat; Lanes 3 and 6, two F1 offspring. (C) Southern blot analysis of on two offspring identified targeted from junction PCR. WT, wild-type goat; Lanes 3 and 6, F1 offspring.
(DOC)

S3 Fig Cell morphology of goat fetal fibroblasts and goat ear fibroblasts. (A) GFFs and GEFs primary cell morphology before transfection. (B) GFF and GEF cell clone morphology after G418 selection. Scale bars represent 100 μm.
(DOC)

S1 Table Primers used to test for gene targeting. (DOC)

S2 Table Gene targeting in goat fibroblasts using TALEN-encoding mRNAs. (DOC)

S3 Table Sequencing results of gene targeted clones in nine potential off-target sites. The potential binding sites of TALENs were in uppercase and the spacers were in lowercase. The mismatches in off-target sites were highlighted in yellow.
(DOC)

S4 Table Summary of BLG-targeted goats. (DOC)

S5 Table Germline transmission of BLG-targeted modification. (DOC)
