# Supplementary material for: Targeting Human α-Lactalbumin Gene Insertion into the Goat β-Lactoglobulin Locus by TALEN-Mediated Homologous Recombination
Source: PLoS One. 2016 Jun 3;11(6):e0156636. doi: 10.1371/journal.pone.0156636 (PMC4892491; doi:10.1371/journal.pone.0156636)
Supplement: S1 Fig — A donor plasmid was created corresponding to the cleavage location of the TALEN pairs, and each side carried approximately 730-bp regions of homology to the BLG sequence astride the cleavage site. (DOC) [file pone.0156636.s001.doc]

**
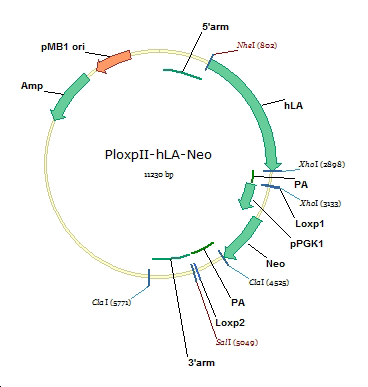
**

**S1 Fig. Vector map of the gene-targeting vector PLoxpII-hLA-neo.** A donor plasmid was created corresponding to the cleavage location of the TALEN pairs, and each side carried approximately 730-bp regions of homology to the BLG sequence astride the cleavage site.
