# Supplementary material for: Targeting Human α-Lactalbumin Gene Insertion into the Goat β-Lactoglobulin Locus by TALEN-Mediated Homologous Recombination
Source: PLoS One. 2016 Jun 3;11(6):e0156636. doi: 10.1371/journal.pone.0156636 (PMC4892491; doi:10.1371/journal.pone.0156636)
Supplement: S2 Fig — (A) 5’Junction PCR analysis on seven F1 offspring. WT, wild-type goat; Lanes 1–7, seven F1 offspring. (B) 3’Junction PCR on two offspring identified targeted from 5’junction PCR. WT, wild-type goat; Lanes 3 and 6, two F1 offspring. (C) Southern blot analysis of on two offspring identified targeted from junction PCR. WT, wild-type goat; Lanes 3 and 6, F1 offspring. (DOC) [file pone.0156636.s002.doc]

**
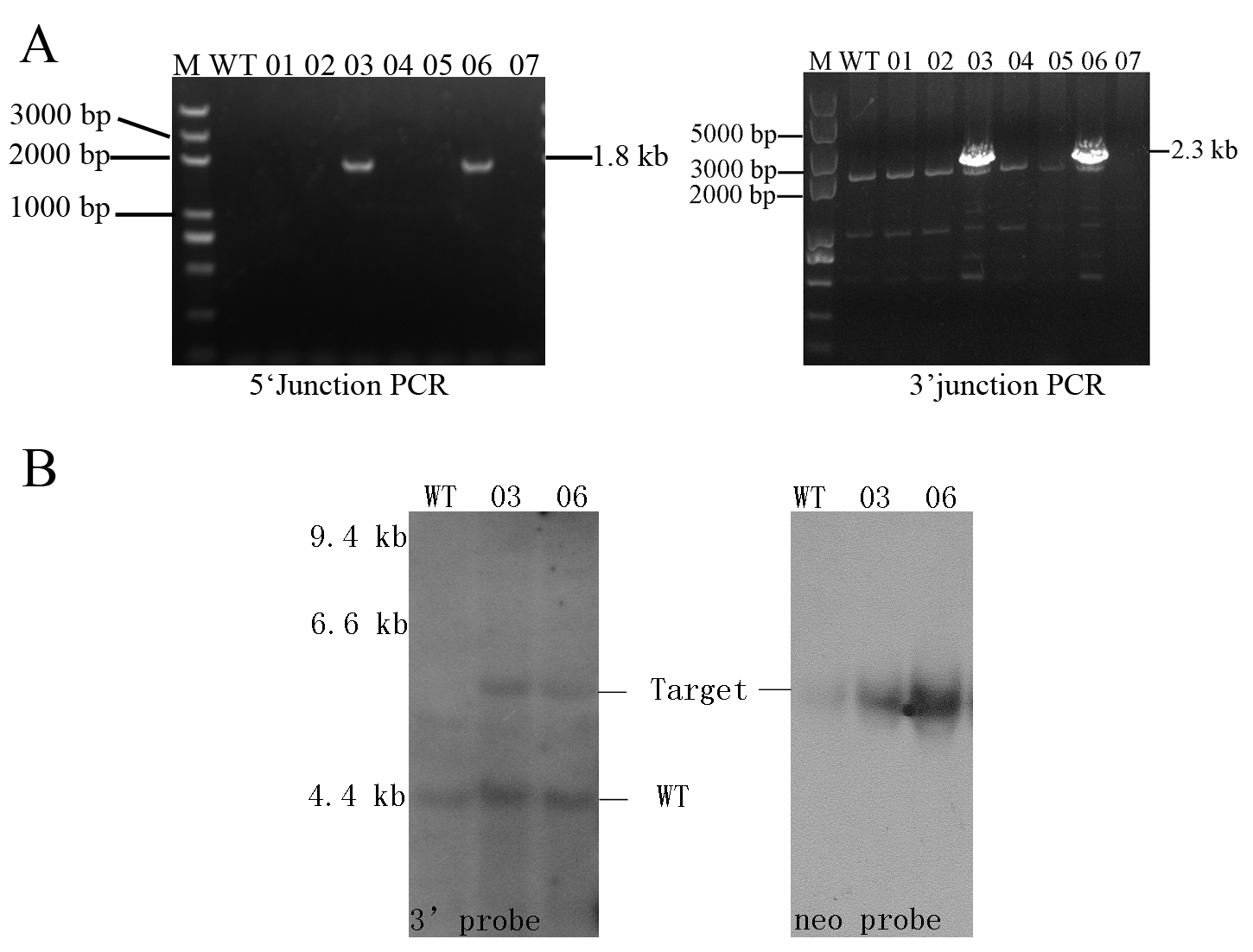
**

**S2 Fig. Analysis of F1 offspring.** (A) 5’Junction PCR analysis on seven F1 offspring. WT, wild-type goat; lanes 1-7, F1 offspring. (B) 3’Junction PCR on seven offspring. WT, wild-type goat; Lanes1-7, F1 offspring. (C) Southern blot analysis on two offspring identified targeted from junction PCR. WT, wild-type goat; Lanes 3 and 6, F1 offspring.
