# Supplementary material for: Targeting Human α-Lactalbumin Gene Insertion into the Goat β-Lactoglobulin Locus by TALEN-Mediated Homologous Recombination
Source: PLoS One. 2016 Jun 3;11(6):e0156636. doi: 10.1371/journal.pone.0156636 (PMC4892491; doi:10.1371/journal.pone.0156636)
Supplement: S3 Fig — (A) GFFs and GEFs primary cell morphology before transfection. (B) GFF and GEF cell clone morphology after G418 selection. Scale bars represent 100 μm. (DOC) [file pone.0156636.s003.doc]

**
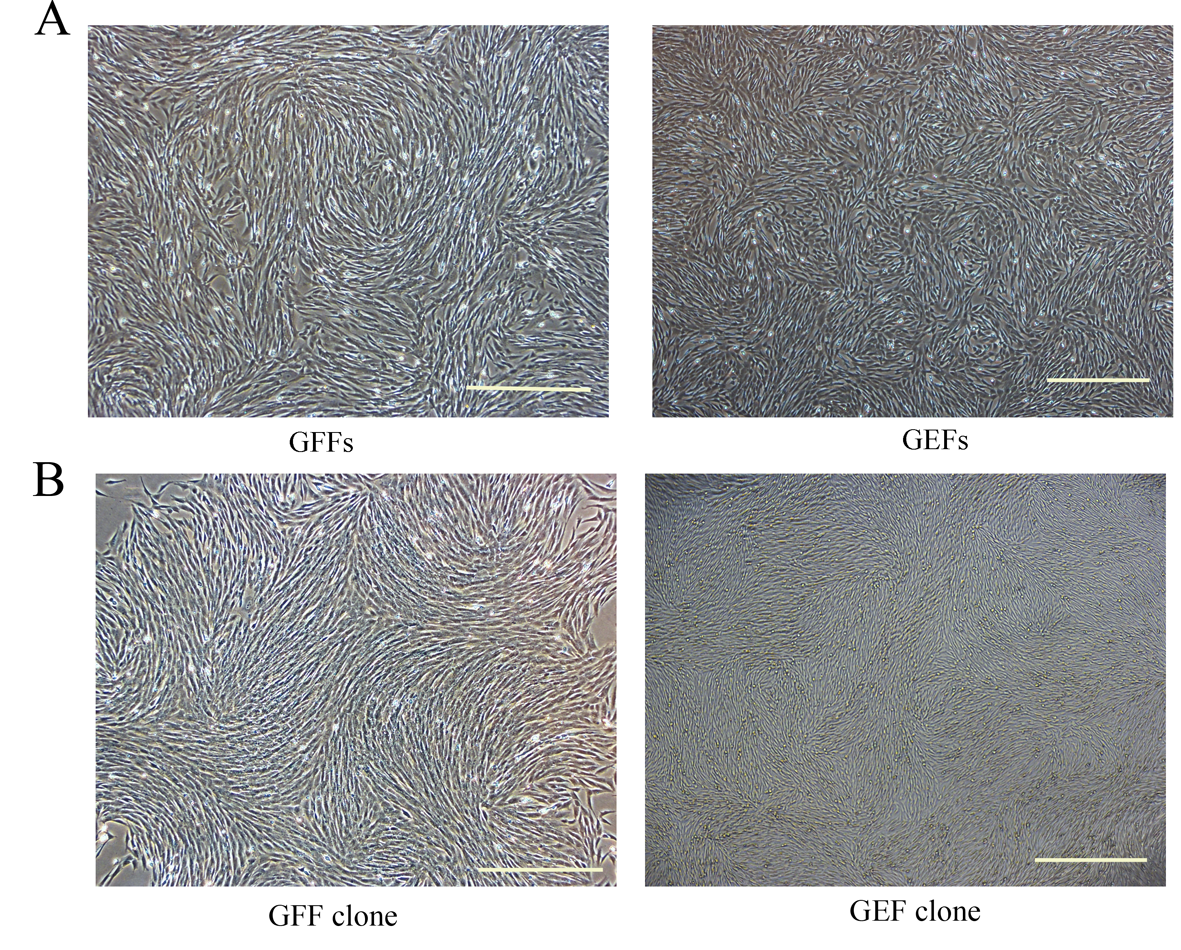
**

**S3 Fig. Cell morphology of goat fetal fibroblasts and goat ear fibroblasts.** (A) GFFs and GEFs primary cell morphology before transfection. (B) GFF and GEF cell clone morphology after G418 selection.Scale bars in two pictures represent 100 mm.
