# Supplementary material for: Targeting Human α-Lactalbumin Gene Insertion into the Goat β-Lactoglobulin Locus by TALEN-Mediated Homologous Recombination
Source: PLoS One. 2016 Jun 3;11(6):e0156636. doi: 10.1371/journal.pone.0156636 (PMC4892491; doi:10.1371/journal.pone.0156636)
Supplement: S1 Table — (DOC) [file pone.0156636.s004.doc]

**S1 Table. Primers used to test for gene targeting.**

| Primer name | Primer sequence | Reaction conditions of PCR | Product size ( bp ) |
| --- | --- | --- | --- |
| B51 | 5’-acagtcaccaacagtctctccgg-3’ | 58°C | 1,800 |
| B52 | 5’-tgatggctcacgcttgtaatccc-3’ |  |  |
| B31 | 5’-tcgaccaccaagcgaaacatcg-3’ | 62°C | 2,300 |
| B32 | 5’-acacacaggcacccccaaaagg-3’ |  |  |
| LRF | 5’-TGACCCAGAGTCCAGACACCC-3’ | 68°C | 4,700 |
| LRR | 5’-CCCTAGCTGACTGATGCGAAC-3’ |  |  |
| Bc-F | 5’-GGGACTTGGTACTCCTTGGCTAT-3’ | 60°C | 200 |
| Bc-R | 5’-ACCGCAGGGATCTTGGTTTT-3’ |  |  |
| Hc-F | 5’-TCGCTTTGCCTGAATTGATCTGTA-3’ | 61°C | 265 |
| Hc-R | 5’-GCTTTATGGGCCAACCAGTAGTCA-3’ |  |  |
