# Supplementary material for: Targeting Human α-Lactalbumin Gene Insertion into the Goat β-Lactoglobulin Locus by TALEN-Mediated Homologous Recombination
Source: PLoS One. 2016 Jun 3;11(6):e0156636. doi: 10.1371/journal.pone.0156636 (PMC4892491; doi:10.1371/journal.pone.0156636)
Supplement: S2 Table — (DOC) [file pone.0156636.s005.doc]

**S2 Table. Gene targeting in goat fibroblasts using TALEN-encoding mRNAs**

| Cells lines  (sex) | Clones picked | Junction(3’and 5’) PCR^+^ clones | LR-PCR^+^  clones | Targeting efficiency (%) |
| --- | --- | --- | --- | --- |
| GEF1 (F) | 260 | 22 | 22 | 8.5 |
| GEF9 (F) | 230 | 23 | 20 | 8.7 |
| GFF3 (F) | 282 | 26 | 26 | 9.3 |
| GFF8 (F) | 260 | 22 | 18 | 6.9 |
| total | 1032 | 93 | 86 | 8.35 |
