# Supplementary material for: Targeting Human α-Lactalbumin Gene Insertion into the Goat β-Lactoglobulin Locus by TALEN-Mediated Homologous Recombination
Source: PLoS One. 2016 Jun 3;11(6):e0156636. doi: 10.1371/journal.pone.0156636 (PMC4892491; doi:10.1371/journal.pone.0156636)
Supplement: S3 Table — The potential binding sites of TALENs were in uppercase and the spacers were in lowercase. The mismatches in off-target sites were highlighted in yellow. (DOC) [file pone.0156636.s006.doc]

## S3 Table. Sequencing results of gene targeted clones in nine potential off-target sites. The potential binding sites of TALENs were in uppercase and the spacers were in lowercase. The mismatches in off-target sites were highlighted in yellow.

| Chr. No. | Sequence(5’ to 3’) | Mismatch | | Spacer(bp) | Detection fragment  (bp) | Mutation clones No. |
| --- | --- | --- | --- | --- | --- | --- |
|  |  | Left | Right |  |  |  |
| 11 (target) | TCCAGGCCATCATCGTcacccagaccatgaaTCCGGACCTGTAGGTCT | 16 | 17 | 15 | - | - |
| 1 | TCCAGGCCAAAATGGCtctcccatcctccaACCATACCTTGAGACCT | 4/16 | 7/17 | 14 | 382 | 0 |
| 2 | CCCAGACCAGCATCAAtgtgtcactgCCCTGAACAGTGGCTCT | 5/16 | 6/17 | 10 | 426 | 0 |
| 10 | ACCAGACCATCTTACCtgcctcctgagaaatAACACACGTCTAGTTCT | 6/16 | 7/17 | 15 | 453 | 0 |
| 18 | TCCAGCACACCACCGCctgagtcaccatgtTCAGGACAGCCAGACTC | 4/16 | 7/17 | 16 | 424 | 0 |
| 26 | CCCAGACCTTAATCCCattattagaacaaTCCGACTCTAAGGTTCT | 6/16 | 6/17 | 12 | 434 | 0 |
| 29 | TCCAGGACAGCATCCTgatagtctatccaacagcctttCCAGAACCTTCAGAACT | 3/16 | 7/17 | 22 | 323 | 0 |
| X | TACCGGCAATCATCTCatggttgttcctggcttccACCAGAACTTACGGTCC | 4/16 | 7/17 | 19 | 366 | 0 |
| Genomic scaffold 1185 | TCTACACCATCACTGCtcccatattcaaccctcACTAGACATGTAACTCT | 6/16 | 6/17 | 17 | 296 | 0 |
