# Supplementary material for: Targeting Human α-Lactalbumin Gene Insertion into the Goat β-Lactoglobulin Locus by TALEN-Mediated Homologous Recombination
Source: PLoS One. 2016 Jun 3;11(6):e0156636. doi: 10.1371/journal.pone.0156636 (PMC4892491; doi:10.1371/journal.pone.0156636)
Supplement: S4 Table — (DOC) [file pone.0156636.s007.doc]

**S4 Table.** Summary of BLG-targeted goats

| Donor cell clones (sex) | Founders | Gestation length (day) | Birth weight  (kg) |
| --- | --- | --- | --- |
| GEF-21 (F) | 02 | 151 | 2.6 |
|  | 03 | 151 | 2.4 |
| GEF-94 (F) | 04 | 153 | 3.5 |
| GEF-97 (F) | 05 | 150 | 3.2 |
|  | 06 | 152 | 3.4 |
| GFF-31 (F) | 01 | 157 | 3.7 |
