# Supplementary material for: Targeting Human α-Lactalbumin Gene Insertion into the Goat β-Lactoglobulin Locus by TALEN-Mediated Homologous Recombination
Source: PLoS One. 2016 Jun 3;11(6):e0156636. doi: 10.1371/journal.pone.0156636 (PMC4892491; doi:10.1371/journal.pone.0156636)
Supplement: S5 Table — (DOC) [file pone.0156636.s008.doc]

**S5 Table. Germline transmission of BLG-targeted modification**

| F0(♂) | F0(♀) | Genotypes of F1 | |
| --- | --- | --- | --- |
|  |  | BLG^+/+^ (Wild-type) | BLG^hLA/+^ |
| BLG^+/+^ (Wild-type) | 02 | 2 | 0 |
|  | 03 | 0 | 1 |
|  | 04 | 2 | 0 |
|  | 05 | 0 | 1 |
|  | 06 | 1 | 0 |
